# Potential Causes of Increased Vocalisation in Elderly Cats with Cognitive Dysfunction Syndrome as Assessed by Their Owners

**DOI:** 10.3390/ani10061092

**Published:** 2020-06-24

**Authors:** Petra Černá, Hannah Gardiner, Lorena Sordo, Camilla Tørnqvist-Johnsen, Danièlle A. Gunn-Moore

**Affiliations:** 1 The Royal (Dick) School of Veterinary Studies and The Roslin Institute, University of Edinburgh, Edinburgh EH25 9RG, UK; lorena.sordo@ed.ac.uk (L.S.); C.T.Johnsen@sms.ed.ac.uk (C.T.-J.); danielle.gunn-moore@ed.ac.uk (D.A.G.-M.); 2 Small Animal Clinic, The University of Veterinary and Pharmaceutical Sciences Brno, 251 61 Brno, Czech Republic; 3 The Beaumont Veterinary Group—Kidlington branch, Kidlington OX5 1EA, UK; hannah@whynotweb.plus.com

**Keywords:** feline, dementia, CDS, geriatric, crying

## Abstract

**Simple Summary:**

Feline cognitive dysfunction syndrome (CDS) is an age-related cognitive deterioration that presents with specific behavioural changes that after thorough investigation cannot be attributed to any other medical condition. Inappropriate vocalisation, especially at night, is one of the behavioural changes in cats with CDS; however, no studies have investigated the potential causes of this distressing behaviour. In this study, owners of cats diagnosed with CDS that presented with increased vocalisation were invited to complete an online survey. The survey consisted of 28 questions including the cat’s signalment, its medical history, and questions pertaining to the owner’s perception of what motivated their cat’s increased vocalisation. Out of 37 responses, the majority of owners reported that the main cause of their cat’s vocalisation appeared to be disorientation (40.5%) or attention seeking (40.5%), followed by seeking a resource such as food (16.2%), and pain (2.7%). Moreover, the majority of owners (64.8%) believed there was >1 cause of their cat’s increased vocalisation. It is important to understand these behavioural changes in cats, especially from the owners´ perspective, as they develop with increasing age and can be distressing to the cat and its owners, potentially influencing their relationship.

**Abstract:**

The objectives of this study were to explore owner perception of the causes of increased vocalisation in cats diagnosed with cognitive dysfunction syndrome (CDS) and consider what impact this vocalisation may have on the cat’s household. Owners of cats diagnosed with CDS that presented with increased vocalisation were invited to complete an online survey. The survey consisted of 28 questions including the cat’s signalment, its medical history, and questions pertaining to the owner’s perception of what motivated their cat´s increased vocalisation. This was determined by looking at the cat’s behaviour when vocalising, where it was looking when it was vocalising, and if the vocalisation stopped when the owner interacted with it, e.g., petting or feeding it. The owners were also asked how stressful they found their cat’s vocalisation. There were 37 responses. The majority of owners reported that the main cause of their cat’s vocalisation appeared to be disorientation (40.5%) or attention seeking (40.5%). Seeking a resource such as food was reported in 16.2%, and pain was perceived to be the cause in only 2.7% of cats. However, the majority of owners (64.8%) believed there was >1 cause of their cat’s increased vocalisation. Importantly, when owners were asked how stressful they found their cat’s increased vocalisation, 40.5% scored ≥3 (where 1 = not stressful; 5 = significantly stressful). This study provides novel insight into owner perception of feline CDS, as well as potential causes for increased vocalisation; this will allow veterinarians to better advise owners on how to manage their cat with CDS.

## 1. Introduction

Feline cognitive dysfunction syndrome (CDS) describes the age-related cognitive deterioration that presents with specific behavioural changes, which, after thorough investigation, cannot be attributed to any other medical condition. The behavioural changes of CDS in cats are summarised by the acronym VISHDAAL [1] and include, but are not limited to: increased Vocalisation, particularly at night, altered Interaction with the family (particularly attention seeking), and House-soiling (Table 1) [2,3,4,5,6]. In contrast, the acronym DISHAAL has been used to refer to the main behavioural changes seen in dogs with CDS, including Disorientation, changes in social Interactions, Sleep-wake cycle alterations, House-soiling, changes in Activity level, Anxiety, and Learning and memory deficits [7]. This acronym has previously been used to summarise the behavioural signs of CDS in cats [7]; however, vocalisation was not included. Given that this is the predominant sign, a new acronym was needed [1]. It is important to understand these behavioural changes in cats, especially from the owners´ perspective, as they can change with increasing age and can influence the relationship between the cat and its owners.

The syndrome of CDS in cats and dogs has been compared with Alzheimer’s disease (AD) in people. All three conditions present with a specific range of behavioural changes (albeit the behaviours differ between the species) and are usually seen with advancing age; in cats, mainly those 11 years and older [6,7,8,9].

They are typically associated with significant neuronal degeneration and the loss of grey and white matter, resulting in brain atrophy, which particularly affects the cerebral cortex where certain proteins often accumulate (in particular, β-amyloid within the neuropil and hyperphosphorylated tau within neurons) [8,9,10]. As with AD, the pathophysiology of CDS is still largely unknown, although compromised cerebral blood flow and chronic free radical damage are believed to be important in both conditions [6,11]. Studies to date have demonstrated vascular changes within the brain of old cats and dogs, including a decrease in cerebral blood flow, arteriosclerosis and the presence of microvascular haemorrhages [4,6,12]. Compromised blood flow and hypoxia due to associated diseases, such as hypertension or heart disease, may also negatively affect the brains of cats and dogs with CDS and people with AD. However, while there are many similarities between CDS and AD, they have not yet been shown to equate to the same disease.

As veterinary care is improving worldwide, the life expectancy of pet cats is increasing. In the US, in 2011, approximately 20% of pet cats were 11 years of age or older [13]; and a 2016 study of cases presenting to veterinary clinics across the UK had a median age of 6.2 years, with cats over 8 years of age representing over 40% of all feline consultations [14]. Many older cats develop behavioural problems (Table 1), often associated with undiagnosed CDS; this has been estimated to affect 28–36% of cats aged 7–14 years, 50% aged 15 years or older, and 88% aged 16–19 years [6,15,16]. One study found excessive vocalisation and aimless activity to be the most commonly reported problems in the oldest age group [6]. Unfortunately, CDS is often underdiagnosed, as clinical signs may be considered “normal” aging changes in elderly pets.

It is important for veterinary professionals to recognise the clinical signs of feline CDS and appreciate that this is a diagnosis of exclusion. Many other diseases can cause behavioural changes that are very similar to those caused by CDS. Hypertension, hyperthyroidism, feline immunodeficiency virus (FIV) infection, and recrudescent toxoplasmosis in particular can present with identical behavioural changes [6,9,17,18,19,20]. While additional clinical signs would usually suggest that one of these other diseases was more likely, e.g., hyphaemia or retinal haemorrhage with hypertension, or weight loss with hyperthyroidism, these other signs are not always present, so a full investigation is always needed. [9,21,22]. Cognitive decline due to FIV-associated neuroinflammation compares with HIV-associated neurocognitive dysfunction (HAND) in people, where the clinical signs can include abnormal stereotypic behaviours, aggression, changes in sleep architecture, and impaired cognitive and motor functions [17,18,19,20].

**Figure 1:**
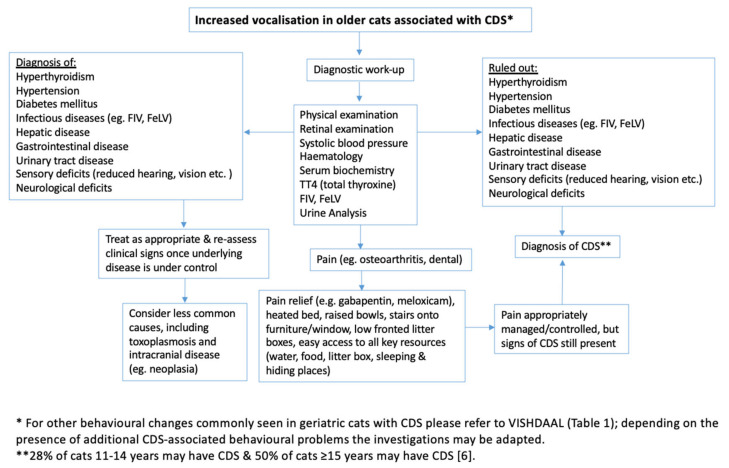
Algorithm for diagnosis of Cognitive dysfunction syndrome (CDS). *FIV*—feline immunodeficiency virus, *FeLV*—feline leukemia virus.

The clinical investigation should be thorough (Figure 1). Physical examination (including a retinal examination looking for signs of hypertension or inflammation) should be followed by measurement of systolic blood pressure (as per ACVIM guidelines) [21]. Systolic blood pressure should always be measured prior to blood draw to avoid situational hypertension [21], and to prevent potentially fatal cerebrovascular accident if the cat is severely hypertensive. This is then followed by haematology, serum biochemistry with electrolytes and serum thyroxine concentration, and complete urine analysis. Cats should also be assessed for potential infectious diseases (e.g., FIV, feline leukaemia virus [FeLV], and toxoplasmosis), particularly if they have outdoor access. While performing a complete neurological and orthopaedic examination can be challenging, especially in elderly cats, the essential requirements are watching the cat walk around the consultation room where obstacles have to be navigated (e.g., their cat basket and the waste bin), plus checking that they can see and hear [9]. Further investigations, such as evaluation of parenchymal brain disease or true behavioural disorders likely require referral, which should be offered. 

It is important that clinical signs compatible with CDS in cats are investigated. This is because there are interventions that may reduce the CDS behaviours (even though there is no cure), other medical problems may also be found that warrant intervention and, regardless of their underlying cause, the behavioural changes can have a significant impact on the owners´ quality of life. The aim of this study was to report the apparent causes of increased vocalisation in cats with CDS as perceived by the cat’s owners.

## 2. Materials and Methods

The cats recruited for this study had previously been involved in a double-blinded placebo-controlled trial for the treatment of CDS with telmisartan. Prior to inclusion in that study, each owner was required to complete a questionnaire (Table S1) to determine that their cat had significant signs of CDS.

Other causes of behavioural change were ruled out prior to inclusion in the initial study. This involved a full physical examination (including retinal examination), systolic blood pressure assessment, haematology, serum biochemistry (including assessment of thyroxine concentration and electrolytes), plus FIV and FeLV testing. Cats with stable IRIS (International Renal Interest Society) grade 1 and 2 chronic kidney disease were allowed in the study. Any cat with systemic disease (e.g., diabetes mellitus, hyperthyroidism, chronic gastrointestinal disease, etc.) had to be on treatment and stable for at least three months prior to inclusion. While *Toxoplasma gondii* serology was not assessed, all cats had full retinal examinations to look for inflammatory (or hypertensive) retinopathy. Additional exclusion criteria included acute behavioural change, episodic lateralizing signs or neurological deficits. Advanced imaging with CT or MRI to rule out intracranial disease (e.g., brain tumours) was not performed.

The cats in the initial study that had increased vocalisation as part of their CDS were invited to take part in the current study, which was performed approximately three months after the conclusion of the initial study.

For the current study, the cat’s owners were sent a questionnaire (Supplementary material 2) composed of 28 questions. It included questions about the cat’s signalment and medical history, and information about the increased vocalisation (when it started, and whether it occurred during the day, at night, or both). It also asked if any other behavioural changes were present, especially those associated with CDS. To try to determine possible motivation for or cause of the increased vocalisation, the owners were asked to notice the “major” behaviour(s) of their cat when it was vocalising:

(i) if it looked directly at them and stopped vocalising when they interacted with it, stroked it, picked it up, etc.—this suggested ***attention seeking***
(ii) if it looked at them, then ran to the food bowl—this suggested the cat was seeking a particular resource, in this case ***food***
(iii) if it was on its own, not looking at them or a particular resource, and appeared confused—this suggested ***disorientation***
(iv) if it appeared to be sore when it moved or was touched—this suggested ***pain***.

The questionnaire also asked the owners how their cat’s increased vocalisation was affecting them. Owners were asked how stressful their cat’s increased vocalisation was on a scale of 1–5, where 1 = not stressful at all and 5 = significant source of stress. Where the cat was deceased, owners were asked to reply as if for their cat’s last few months of life. They were asked multiple-choice questions about how the vocalisation affected their household and if with increasing age they noticed more behavioural signs associated with CDS (as per VISHDAAL).

The results of the questionnaire were exported to an excel document and Minitab software was used for the statistical analysis and production of graphs. Fisher’s exact test was used to determine associations between categorical variables and a *p*-value of less than 0.05 was considered indicative of a statistically significant difference. Spearman’s Rank correlation coefficient was used to summarise the strength and direction of a relationship between two variables (e.g., the number of CDS signs and age of cat, and the number of CDS signs and the impact on the owner).

Both studies were approved by the Veterinary Ethical Review Committee (VERC) and the Human Ethical Review Committee (HERC) at the Royal (Dick) School of Veterinary Studies.

## 3. Results

The survey was open from January 2018 to August 2018; 37 cats with CDS were recruited from 37 households. All had been diagnosed with significant CDS, presenting with increased vocalisation as part of their clinical picture.

All 37 cats had an unremarkable physical examination, facial symmetry and were able to walk around the consultation room without showing lateralizing signs or neurological deficits. Retinal examinations revealed no evidence of inflammatory or hypertensive retinopathy. The cats were normotensive, had normal serum thyroxine concentrations, plus unremarkable haematology and serum biochemistry (i.e., within the allowed parameters), including electrolytes. The cats were FeLV and FIV negative.

The median age of the cats was 15.9 years old (range 10–23). Six of the cats were deceased at the time of this survey; they had been euthanised because of progressive dementia following initial contact or they had developed significant age-related disease. Most of the cats were neutered females (67.6%, *n* = 25); the rest were neutered males (32.4%, *n* = 12). There were significantly more females than males (*p* = 0.005). The most common breed was the Domestic Shorthair (70.3%, *n* = 26), followed by the British Shorthair (10.8%, *n* = 4), Domestic Longhair (8.1%, *n* = 3), Bengal (5.4%, *n* = 2), Siamese (2.7%, *n* = 1) and Persian (2.7%, *n* = 1) cats.

Other than CDS, the most common health problems were osteoarthritis (24.3%; *n* = 9), hyperthyroidism (13.5%; *n* = 5), chronic kidney disease (10.8%; *n* = 4), asthma (5.4%; *n* = 2), diabetes mellitus (5.4%; *n* = 2), hypertension (5.4%; *n* = 2), gastrointestinal small cell lymphoma (2.7%; *n* = 1) and chronic cystitis (2.7%; *n* = 1).

The owners were asked if an initial triggering event had led to their cats increased vocalisation (Table 2).

**Figure 2:**
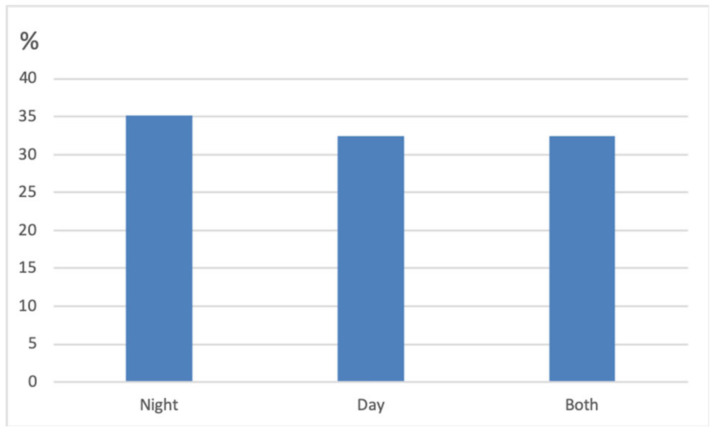
Bar chart illustrating the time of the day the cats most commonly vocalised.

When asked if the cats vocalised at night or during the day, owners reported that 35.1% (*n* = 13) of cats vocalised mostly at night, 34.4% (*n* = 12) vocalised mostly during the day, and 32.4% (*n* = 12) vocalised during both day and night. Fisher’s exact tests showed no statistically significant difference between groups (Figure 2).

**Figure 3:**
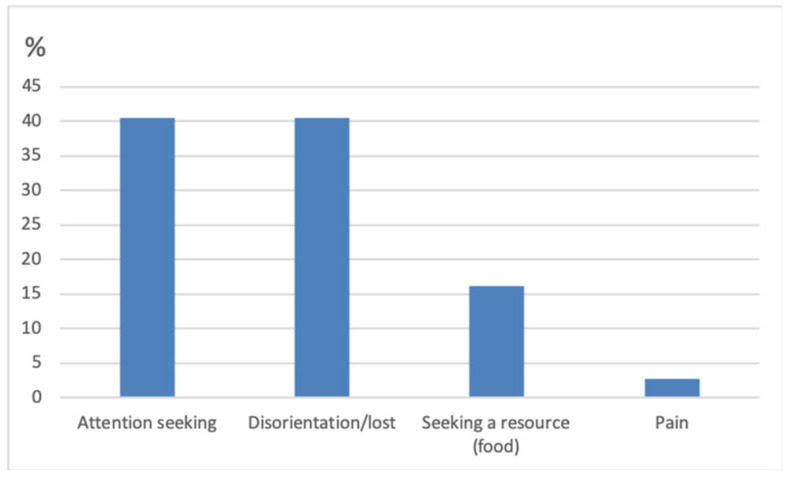
Bar chart illustrating the owners perceived main cause of vocalisation.

When asked about the “major” behaviour and hence likely motivation for their cat’s increased vocalisation, most owners reported that the main cause appeared to be attention seeking (40.5%; *n* = 15) or disorientation (40.5%; *n* = 15) (Figure 3). Seeking a resource such as food was reported by 16.2% of owners. Pain appeared to be the main cause in only 2.7% (*n* = 2) of cats. In most cases (64.8%; *n* = 24) owners believed there to be more than one cause behind their cat’s vocalisation.

A number of other behaviours were associated with the “major” behaviours. For example, behavioural signs associated with attention seeking included increased affection (e.g., wanting to be picked up or petted) (67.6%; *n* = 25), maintained eye contact when vocalising (51.4%; *n* = 19), and the vocalisation stopped after attention from the owner, particularly positive physical contact (43.2%; *n* = 16). For cats that appeared disoriented or lost, owners reported the cat vocalising in a separate room (78.4%; *n* = 29) and aimlessly wandering while vocalising (64.9%; *n* = 24). When seeking a resource (i.e., food) vocalisation occurred at mealtimes (43.2%; *n* = 16) or despite already having eaten (43.2%; *n* = 16).

When asked if, with increasing age, did their cats have more behavioural signs associated with CDS (as per VISHDAAL): Spearman’s rank tests showed a positive trend between the number of CDS signs and the age of cat, although this was not statistically significant.

**Figure 4:**
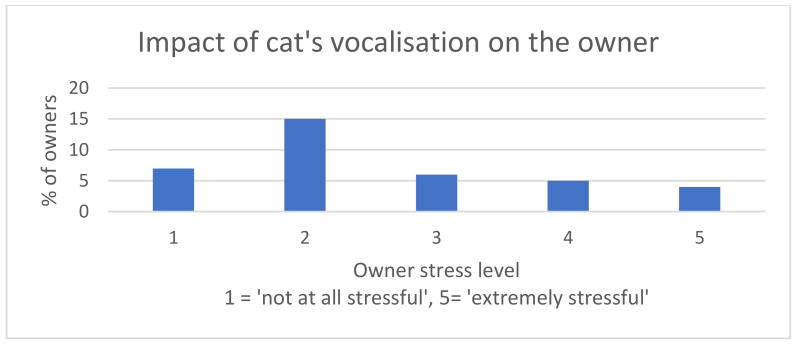
Bar chart summarising the owners’ responses when answering the question of how stressful they found their cat’s excessive vocalisation.

When owners were asked how stressful their cat’s increased vocalisation had been for them, 40.5% responded with a score of 3 or greater, although the most common response was a score of 2 (40.5%; *n* = 15) (Figure 4). For a multiple-choice question, asking how the vocalisation had affected the household, more than half of the owners (62%) selected that they were “occasionally” or “regularly” woken up during the night.

**Figure 5:**
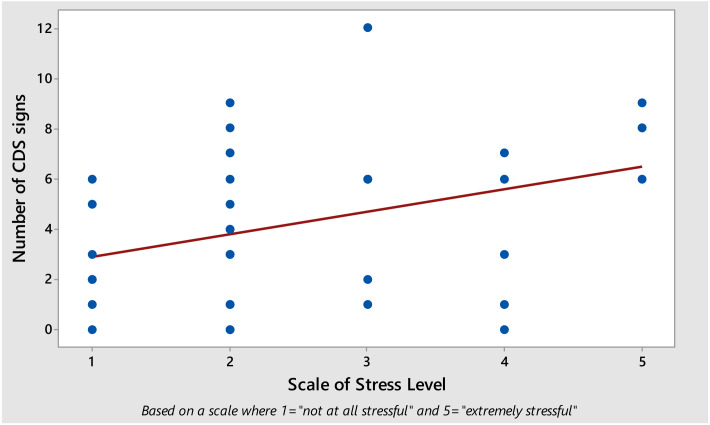
Dot plot illustrating the relationship between the number of clinical signs compatible with CDS and the negative impact on the owner. One dot represents “one owner and their cat” revealing the correlation between the degree of stress to the owner and number of CDS signs in their cat.

Spearman’s rank tests showed a weak to moderate association (r = 0.366, *p* = 0.031) between the number of CDS signs and the impact on the owner, as assessed by the “stress scale” (Figure 5).

In addition to increased vocalisation (which was part of the inclusion criteria), the most common behavioural changes were increased social time with owners (54.0%; *n* = 20), aimless wandering (51.4%; *n* = 19), and staring into space (51.4%; *n* = 19), followed by using less territory outside the house when outside (40.5%; *n* = 15), increased social time with visitors (40.5%; *n* = 15), altered appetite (32.4%; *n* = 12), changes in sleep/wake cycle (29.7%; *n* = 11), decreased grooming (27.0%; *n* = 10), and location related confusion (24.3%; *n* = 9). Other less commonly reported clinical signs were house-soiling (defecation) (16.2%; *n* = 6) and house-soiling (urination) (13.5%; *n* = 5), with only two cats having both urination and defecation reported (5.4%; *n* = 2), increased irritability or aggression (13.5%; *n* = 5), and using less space inside the house (8.1%; *n* = 3). Other behavioural changes included one cat being more playful (2.7%; *n* = 1) and one cat crying in the bath for water and staring at running water but not actually drinking it (2.7%; *n* = 1). 

## 4. Discussion

This study reports the behaviour of 37 cats with CDS and suggests potential causes for their increased vocalisation. The findings generated from the study of our unique population of elderly cats, with their owner’s insight as to associated behaviours that may indicate underlying causes of the vocalisation, provides novel insight into feline CDS. However, before we consider the usefulness of this data in detail, we need to reflect on the limitations of this study. These include the retrospective nature of the data, the small sample size, the possible contribution of other medical conditions (e.g., pain or sensory decline) and that advanced imaging was not performed to rule out intracranial disease such as brain tumours that could be causing similar clinical signs. While all cats had a full physical examination, it is possible that concurrent medical conditions e.g., undiagnosed arthritis, gastrointestinal diseases, etc., may have been playing a role in causing vocalisation. Our best attempts were made to diagnose all concurrent problems, based on a full history, physical examination, blood pressure (BP) and retinal assessment, haematology and serum biochemistry. However, considerably more funding would be needed to undertake more extensive investigation prior to inclusion, ideally including MRI of the head. It would be useful to repeat the study with a larger population; however, given the high costs required to rule out other diseases, this would be challenging. Additionally, it is difficult to justify advanced imaging when patients have gradual behavioural change, no lateralizing signs and no neurological deficits. In addition, our cohort involved dedicated owners who had volunteered for their cats to be enrolled in a clinical trial. These owners may have been more proactive in their cat’s health than many, so they may have been more likely to notice behavioural changes than the general population and, potentially, be more likely to tolerate problem behaviours in their cats. Despite these caveats, we feel the findings generated from this study provides novel insight into owner perception of feline CDS, in particular, the reason(s) behind their increased vocalisation. The recognition of these findings will allow veterinarians to give better advice to owners on how to manage their cat with CDS and to better target the therapy of CDS according to the underlying causes of the cats´ increased vocalisation.

In this study, the median age of the cats was 15.9 years old (range 10–23 years); however, there was no statistically significant correlation between the number of CDS signs and the age of the cat. A previous study looking at 48 otherwise-healthy elderly cats (≥11 years old) that presented for routine health screening and had clinical signs consistent with CDS demonstrated that cats aged 15 years or older had a significantly higher frequency of CDS signs (2.4 per cat) than those 11–14 years old (1.8 per cat) [16]. Further studies are needed to evaluate the progression of CDS, in order to determine if there are increasing numbers of CDS signs with advancing age, and how this affects individual cats.

There were more female cats in the current study, with 67.6% of the cohort being neutered females. The UK pet cat population consists of approximately equal numbers of female and male cats (51.9% female) [17]. While this study had a small sample size, this finding suggests that female cats are potentially more likely to develop CDS; however, further studies are needed to support this finding. In humans, the main risk factors for developing AD are age and gender, with the incidence being higher in women [23,24,25]; 65% of people living with dementia in the UK are female [26]. This predisposition is thought to be mainly due to the increased life expectancy of women, and the association between AD and age. There appears to be no increased life expectancy in female pet cats; a large UK study looking into longevity and mortality found that 50.7% of elderly cat mortalities were female in a single year [27]. While it appears that female cats and people are more likely to develop CDS and AD, respectively, the cause of the female predisposition is unclear.

Increased vocalisation, consisting of excessively loud and/or repetitive verbal utterances and constant requests for attention have been reported in AD [28,29,30]. In people suffering from dementia due to AD or cerebrovascular disease, vocalisation can be triggered by a variety of stimuli, including the physical environment, stress, anxiety, or social factors such as caregiver behaviours [31]. This bears similarities to the cats in the current study, where owner’s observations suggested that vocalisation appeared to be triggered by a need for attention, food, confusion, or pain, highlighting the importance of identifying the potential causes that generate this behaviour. However, since the majority of owners (64.8%) believed there was more than one cause of their cat’s increased vocalisation, simple intervention is not always possible, and multiple approaches may be needed.

It is important to realise that owners may be contributing to the progression of their cats behaviour e.g., by inadvertently reinforcing it by offering food or giving rewards. Moreover, owner emotional responses to their cat’s behaviour, such as distress and frustration, can also have a negative impact on their cat’s state of mind. Owner’s stress (a direct finding in this study), may result as a direct effect from their cat’s vocalisation (including sleep deprivation), as well as their concerns for the well-being of their much loved pet. Owner responses may also compound their cat’s distress if they try to ignore it, or apply any form of punishment to try to stop it crying. It is important to realise that if the cats behavioural need for attention is not met, it may feel distressed and frustrated, negatively affecting their emotional state.

The time of the vocalisation was evenly divided between during the day, at night or both. Those cats that had increased vocalisation at night bear similarities with “sundown syndrome” in human dementia and canine CDS, where there is increased abnormal activity in the late afternoon and early evening [32]. “Sundown syndrome” is linked to the seasons with increased incidence in the autumn or winter months due to a decrease in the duration and amount of sunlight for patients with AD [33]. Additional research is required to see if there is seasonal variation to this behaviour in cats, and to investigate whether the frequency of other CDS signs increases towards evening. However, the difference in natural circadian rhythms between cats, dogs and humans must be considered when evaluating this further [34,35]. Clearly, more work is needed.

In the current study, disorientation and attention seeking appeared to be the most common causes for increased vocalisation. However, the majority of owners felt there were a number of different causes, which was supported by the cats presenting with a variety of altered behaviours. Increased affection was associated with attention seeking in two thirds of the cats in the current study. This finding is supported by a large study evaluating cats of ≥11 years old, from two different time periods (1995 and 2010–2015), with ~2000 respondents [36]. Owners reported an increase in affection from their cats in 52% of cats in 1995, and 36% in 2010–2015 [36]. This increase in need for owners’ affection is believed to result from an augmented dependence on the owners for social support. Alterations in the cats’ relationship with conspecifics, including loss of a conspecific in cases of well bonded cats, may lead to an increased need for social support, especially within multi-cat households. In addition, reduced mobility (arthritis) and/or loss of sensory ability (sight or hearing) compromises effective access to resources, making the cats more dependent on their owners and all of these (immobility, pain, sensory decline and CDS) might initiate or contribute to the attention seeking [36]. The study by Sordo et al. discussed above demonstrated that elderly cats appeared to have reduced motivation to go outdoors, hunt or interact with cats outside the house, and an increased dependency on their owners for company, stimulation, and reassurance [36], which is supported by the current study. Further studies are needed to see if the lack of desire to go outside is caused by CDS alone, or if it is confounded by the reduced ability of older cats to protect themselves, plus difficulties in getting outside due to conditions such as arthritis (particularly when using cat flaps).

It is of note that pain was believed to be the main cause of vocalisation in only 2.7% of cases (i.e., one cat). Perhaps the most common cause of chronic pain in elderly cats is osteoarthritis [37], with neuropathic and cancer pain being poorly assessed [38]. Since >90% of cats aged 12 years or older have evidence of osteoarthritis [39,40], yet it had been diagnosed in only 24.3% of the cats in the current study, this was almost certainly an under-diagnosis, so it may have contributed to more vocalisation than was recognised.

In the developing veterinary climate, with remarkable advances in investigative modalities and treatment options, the importance of behavioural disorders must not be forgotten by veterinary professionals. In addition, behavioural changes can have a marked impact on owners’ quality of life, as demonstrated in the current study, where two thirds of owners were being woken up at night by their cat, and over 80% reported that they found their cat’s behaviour stressful. Perhaps not surprising, the current study found that the owners of the most affected cats were most stressed. Owners were primarily stressed because they were worried about their cat. However, one cat had to be re-homed as the owner was unable to get enough sleep at night. Another reported having to start taking sleeping pills. In contrast, a small proportion of owners reported that they enjoyed the increased affection that occurred with CDS, and this positive effect may outweigh the problem behaviour for some owners. Nevertheless, the stress on owners of having a cat with CDS must be considered.

Veterinarians must educate owners about the prevalence and significance of CDS behaviours and ask owners about them during consultations. Recognising pain can be very challenging in cats due to their ability to hide it well. In our study, only one cat was believed to have increased vocalisation because of pain, which suggests the low awareness of feline pain behaviours by owners rather than the prevalence of this as a cause. Owners appear reticent to seek veterinary attention for these behaviours (particularly vocalisation and house-soiling), possibly because they think they are part of normal ageing, embarrassment that they cannot cope with them, the misconception that nothing can be done to help their cat, the stress of taking their cat to the veterinary clinic, and/or worries that euthanasia will be suggested.

**Figure 6:**
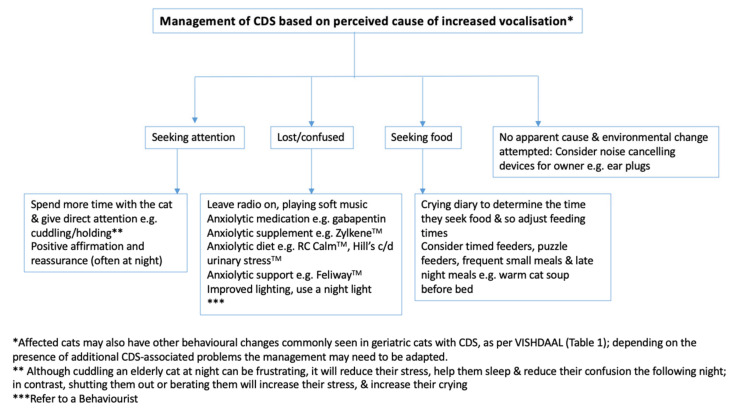
Algorithm for the management of the increased vocalisations associated with Cognitive dysfunction syndrome (CDS), targeting the potential cause, as perceived by the owners. This should be used in conjunction with CDS therapeutics as described below.

Veterinarians must discuss possible environmental modifications and management options that may reduce the impact of CDS on the cat and its family, so improving the cat and the owners’ quality of life. Figure 6 allows the veterinarian to tailor their recommendations for cats with CDS and associated increased vocalisation, focusing on what appears to be causing the crying. However, while simple environmental modifications may help to reduce the vocalisation, additional interventions may be needed to improve the underlying pathophysiology and neuronal dysfunction associated with CDS (see below).

Appropriate interventions for feline CDS include environmental management, food supplements/dietary modification, and drug therapies; this is irrespective of whether vocalisation is part of the clinical presentation or not [9,41]. Environmental enrichment gives mental stimulation, increases activity and leads to survival of neurons in humans [42]. Environmental stimulation (e.g., toys, company, interaction, and food puzzles) and diets enriched with antioxidants act synergistically in dogs with CDS [43]. However, once CDS is severe, environment changes can be stressful, so if changes need to be made, they should be done slowly. It is essential that cats with CDS have access to all key resources (i.e., food, water, litterbox, somewhere to rest/sleep, and either a hiding place/safe haven or access outside) [44] and since arthritis is very common in older cats these resources must be easily reached [9,45,46]. When elderly cats have episodes of confusion and/or crying, they often need much reassurance from their owner (e.g., helping them to settle and calming them down, which often occurs at night). Other suggestions to calm cats with CDS include night-lights, leaving a radio on to play soft music or talk radio, plugging in potentially anxiolytic synthetic pheromones (e.g., Feliway Classic™), and reducing the area the cat has access to so it cannot get lost so easily.

In dogs with CDS, several studies have shown behavioural improvements and reduced amyloid deposition when giving supplements or diets with various combinations of antioxidants, vitamins, essential fatty acids and other potentially useful components (Hill’s b/d; Senilife™; Purina Pro Plan Veterinary Diets Neurocare^®^; CEVA Animal Health; Aktivait™; VetPlus) [41,43,47,48,49,50,51,52,53,54,55,56]. A recent prospective double-blinded placebo controlled clinical study on dogs with CDS showed benefits from a diet with added medium chain triglyceride and Brain Protection Blend in managing clinical signs of CDS [56]. Selegiline (Selgian™; Ceva Anipryl™; Pfizer) and propentofylline (Vivitonin™; MSD Animal Health) are licensed for the management of CDS in dogs, while the nutraceutical S-adenosyl-l-methionine (SAMe) produced increase activity and awareness [56,57,58,59].

There are far fewer feline studies and there are currently no drugs are licensed for the treatment of CDS in cats. Diets show promise, with antioxidants, arginine, B vitamins, fish oil, tocopherols, l-carnitine, vitamin C, beta-carotene, docosa-hexaenoic acid, cysteine and methionine being suggested to have a benefit in cats with CDS, improving brain function of middle-aged and old cats [60,61]. Interestingly, SAMe, also appears to be of some benefit, improving executive function and reduced reversal learning errors but they were ineffective in improving both short and long-term memory [57]. Since Aktivait™ contains alpha-lipoic acid (which is toxic to cats) [62] a feline-safe version has been produced, but not critically evaluated. Selegiline (0.25–1.0 mg/kg PO q24 h) and propentofylline (12.5 mg/cat PO q24 h) have been used ‘off label’ with varying degrees of success [4,41,63,64]. Anxiolytic and antidepressants drugs have also been given (e.g., gabapentin, fluoxetine, buspirone, benzodiazepines), as have nutraceuticals (e.g., Zylkène™; MSD Animal Health); however, critical studies are still needed. Melatonin may help to restore sleep cycles. Telmisartan, an angiotensin receptor blocker (Semintra™; Boehringer Ingelheim) has demonstrated reduced neurodegeneration in rats, and beneficial effects in people with hypertension and AD [65,66,67,68,69]. A study by one of the authors (Prof. Gunn-Moore) evaluating this medication as treatment for cats with CDS is underway.

## 5. Conclusions

The current study adds useful information about a disease that affects a growing number of elderly cats, causing significant distress to the cats and their owners. Increased vocalisation occurred equally throughout the day and night, and most owners thought that the main cause of their cat’s vocalisation was disorientation or attention seeking, with smaller numbers seeking food or crying due to pain. Tailoring management based on these causes may improve the quality of life of both the cats and their owners. The findings from this survey will be used in clinical practice to improve recognition, understanding, and management of CDS in cats and to open up discussion for future studies. Moreover, it is important to highlight the impact that living with a cat with CDS can cause to its owners. More studies are needed to improve our understanding of this important syndrome so we can help our feline patients and their owners.

## Figures and Tables

**Table 1:** Behavioural changes commonly seen in geriatric cats with cognitive dysfunction syndrome (CDS) are summarised by the acronym VISHDAAL [1].

Behavioural Changes Related to CDS (VISHDAAL):
Inappropriate **V**ocalisation, especially at night
Altered social **I**nteraction with the family and/or other pets
Changes in **S**leep/wake patterns
**H**ouse-soiling
Spatial and temporal **D**isorientation e.g., forgetting the location of their litterbox or that they have been fed
Changes in **A**ctivity e.g., aimless wandering **A**nxiety **L**earning and memory deficits

**Table 2:** Initial trigger event that led to increased vocalisation as reported by the owners.

Trigger	Number of Cats
No initial trigger	62.1%; *n* = 23
Moving house	10.8%; *n* = 4
Loss of a sibling	8.1%; *n* = 3
Loss of a family member	8.1%; *n* = 3
New cat/dog in the family	5.4%; *n* = 2
Going to a cattery	2.7%; *n* = 1
Significant veterinary treatment *	2.7%; *n* = 1

* such as dental treatment.

## Supplementary Materials

The following are available at https://www.mdpi.com/2076-2615/10/6/1092/s1. **Table S1. Inclusion Criteria**—The questionnaire each owner had to complete prior to starting the double-blinded placebo-controlled trial. Owners of cats with potential signs of dementia, confusion etc. were asked if they might be interested in joining a double-blind placebo-controlled drug trial. This questionnaire asked whether each of the listed behaviours had changed between now and a few years ago. The number of behaviours that had changed were evaluated and the cats with sufficient inclusion criteria were invited in for assessment (physical examination, blood pressure measurement, blood tests) and if diagnosed with CDS, they were offered a place in the study. All of the owners and cats from the previous study were included for this study. **Supplementary material 2**—The questionnaire for this study (composed of 28 questions).

## References

[1] Sordo L., Gunn-Moore D. (2020). Cognitive dysfunction in cats: Update on neuropathological and behavioural changes plus clinical management. Vet. Rec..

[2] Chapman B., Voith V. (1990). Behavioral problems in old dogs: 26 cases (1984–1987). J. Am. Vet. Med. Assoc..

[3] Ruehl W., Bruyette D., DePaoli A., Cotman C., Head E., Milgram N., Cummings B. (1995). Canine cognitive dysfunction as a model for human age-related cognitive decline, dementia and Alzheimer’s disease: Clinical presentation, cognitive testing, pathology and response to 1-deprenyl therapy. Prog. Brain Res..

[4] Landsberg G., Araujo J.A. (2005). Behavior problems in geriatric pets. Vet. Clin. N. Am.-Small..

[5] Miele A., Sordo L., Gunn-Moore D. (2020). Feline Aging: Promoting Physiologic and Emotional Well-being. Vet. Clin. North. Am. Small. Anim. Pract..

[6] Gunn-Moore D., Moffat K., Christie L.A., Head E. (2007). Cognitive dysfunction and the neurobiology of ageing in cats. J. Small Anim. Pract..

[7] Landsberg G.M., Nichol J., Araujo J.A. (2012). Cognitive dysfunction syndrome: A disease of canine and feline brain aging. Vet. Clin. North Am. Small. Anim. Pract..

[8] Gunn-Moore D.A., McVee J., Bradshaw J.M., Pearson G.R., Head E., Gunn-Moore F.J. (2006). Ageing changes in cat brains demonstrated by β-amyloid and AT8-immunoreactive phosphorylated tau deposits. J. Feline Med. Surg..

[9] Gunn-Moore D.A. (2011). Cognitive dysfunction in cats: Clinical assessment and management. Top. Companion Anim. Med..

[10] Serrano-Pozo A., Frosch M.P., Masliah E., Hyman B.T. (2011). Neuropathological alterations in Alzheimer disease. Cold Spring Harb. Perspect. Med..

[11] Volicer L., Crino P.B. (1990). Involvement of free radicals in dementia of the Alzheimer type: A hypothesis. Neurobiol. Aging.

[12] Dimakopoulos A.C., Mayer R.J. (2002). Aspects of neurodegeneration in the canine brain. J. Nutr..

[13] Bellows J., Center S., Daristotle L., Estrada A.H., Flickinger E.A., Horwitz D.F., Lascelles B.D.X., Lepine A., Perea S., Scherk M. (2016). Evaluating aging in cats: How to determine what is healthy and what is disease. J. Feline Med. Surg..

[14] Sánchez-Vizcaíno F., Noble P.-J.M., Jones P.H., Menacere T., Buchan I., Reynolds S., Dawson S., Gaskell R.M., Everitt S., Radford A.D. (2017). Demographics of dogs, cats, and rabbits attending veterinary practices in Great Britain as recorded in their electronic health records. BMC Vet. Res..

[15] Landsberg G. Behavior problems of older cats. Proceedings of the 135th annual meeting of the American Veterinary Medical Association.

[16] Moffatt K., Landsberg G. (2003). An investigation of the prevalence of clinical signs of cognitive dysfunction syndrome (CDS) in cats. J. Am. Anim. Hosp. Assoc..

[17] Gunn-Moore D.A., Pearson G.R., Harbour D.A., Whiting C.V. (1996). Encephalitis associated with giant cells in a cat with naturally occurring feline immunodeficiency virus infection demonstrated by in situ hybridization. Vet. Pathol..

[18] Meeker R.B., Hudson L. (2017). Feline Immunodeficiency Virus Neuropathogenesis: A Model for HIV-Induced CNS Inflammation and Neurodegeneration. J. Vet. Sci..

[19] Joseph J. (2018). Optimizing animal models for HIV-associated CNS dysfunction and CNS reservoir research. J. Neurovirol..

[20] Power C. (2018). Neurologic disease in feline immunodeficiency virus infection: Disease mechanisms and therapeutic interventions for NeuroAIDS. J. Neurovirol..

[21] Acierno M.J., Brown S., Coleman A.E., Jepson R.E., Papich M., Stepien R.L., Syme H.M. (2018). ACVIM consensus statement: Guidelines for the identification, evaluation, and management of systemic hypertension in dogs and cats. J. Vet. Intern. Med..

[22] McLean J.L., Lobetti R.G., Schoeman J.P. (2014). Worldwide prevalence and risk factors for feline hyperthyroidism: A review. J. S. Afr. Vet. Assoc..

[23] Andersen K., Launer L.J., Dewey M.E., Letenneur L., Ott A., Copeland J., Dartigues J.-F., Kragh-Sorensen P., Baldereschi M., Brayne C. (1999). Gender differences in the incidence of AD and vascular dementia: The EURODEM Studies. Neurology.

[24] Carter C.L., Resnick E.M., Mallampalli M., Kalbarczyk A. (2012). Sex and gender differences in Alzheimer’s disease: Recommendations for future research. J. Womens Health.

[25] Mielke M.M., Vemuri P., Rocca W.A. (2014). Clinical epidemiology of Alzheimer’s disease: Assessing sex and gender differences. Clin. Epidemiol..

[26] Prince M., Knapp M., Guerchet M., McCrone P., Prina M., Comas-Herrera A., Wittenberg R., Adelaja B., Hu B., King D. (2014). Dementia UK Update.

[27] O’Neill D.G., Church D.B., McGreevy P.D., Thomson P.C., Brodbelt D.C. (2015). Longevity and mortality of cats attending primary care veterinary practices in England. J. Feline Med. Surg..

[28] Ryan D.P., Tainsh S.M., Kolodny V., Lendrum B.L., Fisher R.H. (1988). Noise-making amongst the elderly in long term care. Gerontologist.

[29] Cohen-Mansfield J., Werner P., Marx M.S. (1990). Screaming in nursing home residents. J. Am. Geriatr. Soc..

[30] Cariaga J., Burgio L., Flynn W., Martin D. (1991). A controlled study of disruptive vocalizations among geriatric residents in nursing homes. J. Am. Geriatr. Soc..

[31] Matteau E., Landreville P., Laplante L., Laplante C. (2003). Disruptive vocalizations: A means to communicate in dementia?. Am. J. Alzheimers Dis. Other Demen..

[32] Schake C. Sundowner Syndrome in Dogs. https://positively.com/contributors/sundowner-syndrome-in-dogs/.

[33] Volicer L., Harper D.G., Manning B.C., Goldstein R., Satlin A. (2001). Sundowning and circadian rhythms in Alzheimer’s disease. Am. J. Psychiatry.

[34] Reinberg A., Ashkenazi I. (2003). Concepts in human biological rhythms. Dialogues Clin. Neurosci..

[35] Moore-Ede M.C., Sulzman F.M., Fuller C.A. (1984). The Clocks that Time Us: Physiology of the Circadian Timing System.

[36] Sordo L., Breheny C., Halls V., Gunn-Moore D. (2020). Behaviour and health of elderly cats in the UK: Changes over 20 years. Vet. Sci..

[37] Epstein M., Rodan I., Griffenhagen G., Kadrlik J., Petty M., Robertson S., Simpson W. (2015). 2015 AAHA/AAFP Pain Management Guidelines for Dogs and Cats. J. Am. Anim. Hosp. Assoc..

[38] Lascelles B.D.X., Brown D.C., Conzemius M.G., Gill M., Oshinsky M.L., Sharkey M. (2019). Measurement of chronic pain in companion animals: Discussions from the Pain in Animals Workshop (PAW) 2017. Vet. J..

[39] Bennett D., Zainal Ariffin S.M., Johnston P. (2012). Osteoarthritis in the cat: 1. how common is it and how easy to recognise?. J. Feline Med. Surg..

[40] Hardie E.M., Roe S.C., Martin F.R. (2002). Radiographic evidence of degenerative joint disease in geriatric cats: 100 cases (1994–1997). J. Am. Anim. Hosp. Assoc..

[41] Landsberg G. (2006). Therapeutic options for cognitive decline in senior pets. J. Am. Anim. Hosp. Assoc..

[42] Galvan V., Bredesen D.E. (2007). Neurogenesis in the adult brain: Implications for Alzheimer’s disease. CNS Neurol. Disord. Drug Targets.

[43] Pop V., Head E., Hill M.-A., Gillen D., Berchtold N.C., Muggenburg B.A., Milgram N.W., Murphy M.P., Cotman C.W. (2010). Synergistic effects of long-term antioxidant diet and behavioral enrichment on β-amyloid load and non-amyloidogenic processing in aged canines. J. Neurosci..

[44] Ellis S.L.H., Rodan I., Carney H.C., Heath S., Rochlitz I., Shearburn L.D., Sundahl E., Westropp J.L. (2013). AAFP and ISFM Feline Environmental Needs Guidelines. J. Feline Med. Surg..

[45] Slingerland L., Hazewinkel H., Meij B., Picavet P., Voorhout G. (2011). Cross-sectional study of the prevalence and clinical features of osteoarthritis in 100 cats. Vet. J..

[46] Lascelles B.D.X. (2010). Feline Degenerative Joint Disease. Vet. Surg..

[47] Ikeda-Douglas C., Zicker S., Estrada J., Jewell D., Milgram N. (2004). Prior experience, antioxidants, and mitochondrial cofactors improve cognitive function in aged beagles. Vet. Ther..

[48] Head E., Zicker S.C. (2004). Nutraceuticals, aging, and cognitive dysfunction. Vet. Clin. North Am. Small Anim. Pract..

[49] Roudebush P., Zicker S.C., Cotman C.W., Milgram N.W., Muggenburg B.A., Head E. (2005). Nutritional management of brain aging in dogs. J. Am. Vet. Med. Assoc..

[50] Heath S.E., Barabas S., Craze P.G. (2007). Nutritional supplementation in cases of canine cognitive dysfunction—A clinical trial. Appl. Anim. Behav. Sci..

[51] Pan Y., Larson B., Araujo J.A., Lau W., De Rivera C., Santana R., Gore A., Milgram N.W. (2010). Dietary supplementation with medium-chain TAG has long-lasting cognition-enhancing effects in aged dogs. Br. J. Nutr..

[52] Fragua V., Lepoudère A., Leray V., Baron C., Araujo J., Nguyen P., Milgram N. (2017). Effects of dietary supplementation with a mixed blueberry and grape extract on working memory in aged beagle dogs. J. Nutr. Sci..

[53] Pan Y., Kennedy A.D., Jönsson T.J., Milgram N.W. (2018). Cognitive enhancement in old dogs from dietary supplementation with a nutrient blend containing arginine, antioxidants, B vitamins and fish oil. Br. J. Nutr..

[54] Araujo J.A., Landsberg G.M., Milgram N.W., Miolo A. (2008). Improvement of short-term memory performance in aged beagles by a nutraceutical supplement containing phosphatidylserine, Ginkgo biloba, vitamin E, and pyridoxine. Can. Vet. J..

[55] Pan Y., Landsberg G., Mougeot I., Kelly S., Xu H., Bhatnagar S., Gardner C.L., Milgram N.W. (2018). Efficacy of a Therapeutic Diet on Dogs With Signs of Cognitive Dysfunction Syndrome (CDS): A Prospective Double Blinded Placebo Controlled Clinical Study. Front. Nutr..

[56] Landsberg G.M., DePorter T., Araujo J.A. (2011). Clinical signs and management of anxiety, sleeplessness, and cognitive dysfunction in the senior pet. Vet. Clin. North Am. Small Anim. Pract..

[57] Araujo J.A., Faubert M.L., Brooks M.L., Landsberg G.M., Lobprise H. (2012). NOVIFIT^®^(NoviSAMe^®^) Tablets Improve Executive Function in Aged Dogs and Cats: Implications for Treatment of Cognitive Dysfunction Syndrome. Int. J. Appl. Res. Vet. M..

[58] Bottiglieri T. (2002). S-Adenosyl-L-methionine (SAMe): From the bench to the bedside—molecular basis of a pleiotrophic molecule. Am. J. Clin. Nutr..

[59] Rème C.-A., Dramard V., Kern L., Hofmans J., Halsberghe C., Mombiela D.V. (2008). Effect of S-adenosylmethionine tablets on the reduction of age-related mental decline in dogs: A double-blinded, placebo-controlled trial. Vet. Ther..

[60] Pan Y., Araujo J.A., Burrows J., de Rivera C., Gore A., Bhatnagar S., Milgram N.W. (2013). Cognitive enhancement in middle-aged and old cats with dietary supplementation with a nutrient blend containing fish oil, B vitamins, antioxidants and arginine. Br. J. Nutr..

[61] Houpt K., Levine E., Landsberg G., Moffat K.S., Zicker S.C., Moffat K.S. (2007). Antioxidant fortified food improves owner perceived behaviour in the aging cat. Proceedings of the ESFM Conference.

[62] Hill A., Werner J., Rogers Q., O’Neill S., Christopher M.M. (2004). Lipoic acid is 10 times more toxic in cats than reported in humans, dogs or rats. J. Anim. Physiol. Anim. Nutr..

[63] Landsberg G.M., Hunthausen W.L., Ackerman L.J., Landsberg G., Hunthausen W., Ackerman L. (2003). Handbook of Behavior Problems of the Dog and Cat.

[64] Studzinski C.M., Araujo J.A., Milgram N.W. (2005). The canine model of human cognitive aging and dementia: Pharmacological validity of the model for assessment of human cognitive-enhancing drugs. Prog. Neuropsychopharmacol. Biol. Psychiatry.

[65] Kume K., Hanyu H., Sakurai H., Takada Y., Onuma T., Iwamoto T. (2012). Effects of telmisartan on cognition and regional cerebral blood flow in hypertensive patients with Alzheimer’s disease. Geriatr. Gerontol. Int..

[66] Wang J., Pang T., Hafko R., Benicky J., Sanchez-Lemus E., Saavedra J.M. (2014). Telmisartan ameliorates glutamate-induced neurotoxicity: Roles of AT1 receptor blockade and PPARγ activation. Neuropharmacology.

[67] Wincewicz D., Braszko J.J. (2014). Telmisartan attenuates cognitive impairment caused by chronic stress in rats. Pharmacol. Rep..

[68] Li W., Zhang J., Lu F., Ma M., Wang J., Suo A., Bai Y., Liu H. (2012). Effects of telmisartan on the level of Aβ1-42, interleukin-1β, tumor necrosis factor α and cognition in hypertensive patients with Alzheimer’s disease. Zhonghua yi xue za zhi.

[69] Pan G., Zhou X., Zhao J. (2014). Effect of Telmisartan on Atrial Fibrillation Recurrences in Patients with Hypertension: A Systematic Review and Meta-Analysis. Cardiovasc. Ther..

