# Supplementary material for: Potential Causes of Increased Vocalisation in Elderly Cats with Cognitive Dysfunction Syndrome as Assessed by Their Owners
Source: Animals (Basel). 2020 Jun 24;10(6):1092. doi: 10.3390/ani10061092 (PMC7341261; doi:10.3390/ani10061092)
Supplement: Supplementary file 1 [file animals-10-01092-s001.zip › Table S1.docx]

**Appendix A**

**Table S1. INCLUSION CRITERIA**

|  | **Weighting** | **Case Number**  **SCS …………** |
| --- | --- | --- |
| Change in weight | 2 |  |
| Appetite | 2 |  |
| Drinking | 3 |  |
| Time spent grooming | 1 |  |
| Time spent sleeping during the day | 1 |  |
| Time spent sleeping at night | 1 |  |
| Vocalisation at night | 1* |  |
| Vocalisation during the day | 1 |  |
| Affection with people in the house | 1* |  |
| Tolerance of handling | 2 |  |
| Aggression towards animals or people | 2 |  |
| Wanting to spend time outdoors | 2 |  |
| Activity levels/time spent playing | 2 |  |
| Tolerance of being left alone | 1* |  |
| Tolerance of other animals in the house | 2 |  |
| Willingness to jump up or down  (including climbing stairs) | 3 |  |
| Aimless activity  (eg pacing, staring into space) | 1 |  |
| Repetitive or compulsive behaviour  (eg grooming, licking inanimate objects) | 1 |  |
| Agitation and restlessness | 2 |  |
| Passing faeces in house outside litter tray | 2 |  |
| Passing urine in house outside litter tray | 2 |  |
| Vomiting (including furballs) | 2 |  |
| Diarrhoea | 3 |  |
| Constipation | 3 |  |
| Vision loss | 3 |  |
| Hearing loss | 3 |  |
| Hair loss or hair thinning | 3 |  |
|  | | |
| TALLY FOR THIS CAT: | |  |

To be included in the trial a cat must score a minimum of:

- one x1* or
- two x 1 or
- four x 2.
