# Supplementary material for: Potential Causes of Increased Vocalisation in Elderly Cats with Cognitive Dysfunction Syndrome as Assessed by Their Owners
Source: Animals (Basel). 2020 Jun 24;10(6):1092. doi: 10.3390/ani10061092 (PMC7341261; doi:10.3390/ani10061092)
Supplement: Supplementary file 1 [file animals-10-01092-s001.zip › Supplementary material 2.doc]

# Supplementary material 2

# SENIOR CAT STUDY

# QUESTIONNAIRE 1

Surname ……………………………………………………………

Address .……………………………………………………………………….

……………………………………………………………………………………

………………………………………Post Code ………………………………

Telephone ………………………………………………………………………

Email ……………………………………………………………………………

Cat’s Name	……………………………………………………………………

Breed ………………………………………	Age ………yrs………months

How long has this cat been living with you? ………yrs………months

Gender: Male □	Male neutered □	Female □	Female neutered □

Does your cat have outdoor access?	Yes □	No □

Does your cat have a litter tray indoors?	Yes □	No □

Has your cat been diagnosed with any medical conditions? Yes □	No □

If yes, please state what …………………………………………………………

Is your cat currently on any medication?	Yes □	No □

If yes, please state what …………………………………………………………

(Please include all food supplements, herbal remedies, over-the-counter tablets or capsules and any medications prescribed by your vet).

Would you be willing to administer a daily food supplement to your cat and answer several questionnaires over the next few months as part of a study into cognitive decline in senior cats?

Yes □	No □

Please answer the questions below by placing a tick in the appropriate column. For each question, think about how your cat used to be and compare this with how he/she is now. If you have noticed a change please indicate how significant you think this change has been.

|  | **Significant decrease** | **Mild decrease** | **No change** | **Mild increase** | **Significant increase** | **Never seen** |
| --- | --- | --- | --- | --- | --- | --- |
| Change in weight |  |  |  |  |  |  |
| Appetite |  |  |  |  |  |  |
| Drinking |  |  |  |  |  |  |
| Time spent grooming |  |  |  |  |  |  |
| Time spent sleeping during the day |  |  |  |  |  |  |
| Time spent sleeping at night |  |  |  |  |  |  |
| Vocalisation at night |  |  |  |  |  |  |
| Vocalisation during the day |  |  |  |  |  |  |
| Affection with people in the house |  |  |  |  |  |  |
| Tolerance of handling |  |  |  |  |  |  |
| Aggression towards animals or people |  |  |  |  |  |  |
| Wanting to spend time outdoors |  |  |  |  |  |  |
| Activity levels/time spent playing |  |  |  |  |  |  |
| Tolerance of being left alone |  |  |  |  |  |  |
| Tolerance of other animals in the house |  |  |  |  |  |  |

|  | **Significant decrease** | **Mild decrease** | **No change** | **Mild increase** | **Significant increase** | **Never seen** |
| --- | --- | --- | --- | --- | --- | --- |
| Willingness to jump up or down  (including climbing stairs) |  |  |  |  |  |  |
| Aimless activity  (eg pacing, staring into space) |  |  |  |  |  |  |
| Repetitive or compulsive behaviour  (eg grooming, licking inanimate objects) |  |  |  |  |  |  |
| Agitation and restlessness |  |  |  |  |  |  |
| Passing faeces in house outside litter tray |  |  |  |  |  |  |
| Passing urine in house outside litter tray |  |  |  |  |  |  |
| Vomiting (including furballs) |  |  |  |  |  |  |
| Diarrhoea |  |  |  |  |  |  |
| Constipation |  |  |  |  |  |  |
| Vision loss |  |  |  |  |  |  |
| Hearing loss |  |  |  |  |  |  |
| Hair loss or hair thinning |  |  |  |  |  |  |

How long ago did you first notice any changes listed above? ...……...………………………………………………………………………

Are there any other problems you think it may be useful for us to know? ……………………………………………………………………

*Thank you for completing this survey. Please return all three pages in the stamped self-address envelope as soon as possible.*

We will be in touch to arrange a full consultation if your cat is eligible to take part in the study.
